# Supplementary material for: Modifying and validating the social responsiveness scale edition 2 for use with deaf children and young people
Source: PLoS One. 2020 Dec 7;15(12):e0243162. doi: 10.1371/journal.pone.0243162 (PMC7721463; doi:10.1371/journal.pone.0243162)
Supplement: S1 Appendix — (DOCX) [file pone.0243162.s001.docx]

|  |  |
| --- | --- |
| 1 | Seems much more fidgety in social situations than when alone |
| 2 | Expressions on his or her face don’t match what he or she is saying or signing |
| 3 | Seems self-confident when interacting with others who can communicate with them |
| 4 | When under stress, he or she shows rigid or inflexible patterns of behaviour that seem odd |
| 5 | Doesn’t recognize when others are trying to take advantage of him or her |
| 6 | Would rather be alone than with others |
| 7 | Is aware that other people may have different thoughts or feelings from his/her own |
| 8 | Behaves in ways that seem strange or bizarre |
| 9 | Seems too dependent on adults |
| 10 | Takes things more literally from other people’s conversations than you would expect for his/her language development |
| 11 | Has good self-confidence in some everyday activities |
| 12 | Is able to communicate his or her feelings to others through words, signs or gestures |
| 13 | Is awkward in turn-taking interactions with peers (for example, doesn’t seem to understand the give-and take in conversations or games) |
| 14 | Is not well coordinated in everyday physical activities |
| 15 | Is able to understand the meaning of other people’s body language and facial expression |
| 16 | Avoids eye contact or has unusual eye contact |
| 17 | Recognises or shows understanding when something is unfair |
| 18 | Makes little attempt to interact with other children (deaf or hearing) or has difficulty making friends, even when trying his or her best |
| 19 | Gets frustrated trying to get ideas across to those who can communicate with them |
| 20 | Shows unusual sensory interests (for example, mouthing or spinning objects) or strange ways of playing with toys |
| 21 | Is able to imitate others in play |
| 22 | Plays appropriately with children in playgroup or class |
| 23 | Does not join group activities unless told to do so |
| 24 | Has more difficulty than other children with changes in his or her routine when they have been clearly informed of the change |
| 25 | Doesn’t seem to mind being different or not on the same wavelength as others |
| 26 | Offers comfort to others when they are sad |
| 27 | Avoids starting social interactions even when peers or adults are using the child’s preferred form of communication |
| 28 | Thinks or talks about the same thing over and over |
| 29 | Other deaf or hearing children may describe him or her as odd or weird |
| 30 | Becomes upset in a situation with lots of things going on even when communication is available to the child |
| 31 | Can't get his or her mind off something once he or she starts thinking about it |
| 32 | Shows age appropriate awareness of personal hygiene |
| 33 | Is awkward with familiar children and adults in a social setting, even when he or she is trying to be polite |
| 34 | Avoids people who want to be emotionally close to him or her |
| 35 | Has trouble keeping up with the flow of a conversation in his/her preferred language. |
| 36 | Has difficulty relating to adults |
| 37 | Has difficulty relating to other children (deaf and hearing) |
| 38 | Responds appropriately to mood changes in others (for example, when a friend's or playmate's mood changes from happy to sad) |
| 39 | Has an unusually narrow range of interests |
| 40 | Is imaginative, good at pretending (without losing touch with reality) |
| 41 | Wanders aimlessly from one activity to another and does not remain engaged in a particular activity |
| 42 | Seems overly sensitive to textures, smells or tastes |
| 43 | Separates easily from caregivers |
| 44 | Doesn't understand how events relate to one another (cause and effect) the way other children his or her age do |
| 45 | Focuses his or her attention to where others are looking |
| 46 | Has overly serious or blank facial expressions |
| 47 | Is too silly or laughs inappropriately |
| 48 | Has a sense of humour, can share in a joke with others |
| 49 | Has one or more exceptional skills |
| 50 | Has repetitive, odd behaviours such as hand flapping or rocking |
| 51 | Responds to clear, direct questions in ways that don’t seem to make any sense or go off subject |
| 52 | Seems to react to people as if they are objects |
| 53 | Knows when he or she is too close to someone or is invading someone's space |
| 54 | Walks in between two people who are talking/signing without acknowledgement |
| 55 | Other children, deaf or hearing, do not like to play with him or her |
| 56 | Concentrates too much on parts of things rather than seeing the whole picture. For example, spins the wheels of toy car but doesn’t play with it as a car, or if asked to describe a film, only focuses on small details rather than the main events |
| 57 | Is suspicious of most people |
| 58 | Is emotionally distant, doesn't show his or her feelings |
| 59 | Is inflexible, has a hard time changing his or her mind |
| 60 | Gives unusual or illogical reasons for doing things |
| 61 | Touches others in an unusual way (for example may stroke a stranger's hair or their clothing) |
| 62 | Is very tense in most social settings |
| 63 | Stares or gazes off into space and is difficult to engage |
| 64 | Does not appear to be aware that they might need to adapt their communication depending on whether they're communicating with a deaf or a hearing person. |
